# Modeling and Automatic Feedback Control of Tremor: Adaptive Estimation of Deep Brain Stimulation

**DOI:** 10.1371/journal.pone.0062888

**Published:** 2013-04-24

**Authors:** Muhammad Rehan, Keum-Shik Hong

**Affiliations:** 1 Department of Electrical Engineering, Pakistan Institute of Engineering and Applied Sciences (PIEAS), Islamabad, Pakistan; 2 Department of Cogno-Mechatronics Engineering and School of Mechanical Engineering, Pusan National University, Geumjeong-gu, Busan, Republic of Korea; Rutgers University, United States of America

## Abstract

This paper discusses modeling and automatic feedback control of (postural and rest) tremor for adaptive-control-methodology-based estimation of deep brain stimulation (DBS) parameters. The simplest linear oscillator-based tremor model, between stimulation amplitude and tremor, is investigated by utilizing input-output knowledge. Further, a nonlinear generalization of the oscillator-based tremor model, useful for derivation of a control strategy involving incorporation of parametric-bound knowledge, is provided. Using the Lyapunov method, a robust adaptive output feedback control law, based on measurement of the tremor signal from the fingers of a patient, is formulated to estimate the stimulation amplitude required to control the tremor. By means of the proposed control strategy, an algorithm is developed for estimation of DBS parameters such as amplitude, frequency and pulse width, which provides a framework for development of an automatic clinical device for control of motor symptoms. The DBS parameter estimation results for the proposed control scheme are verified through numerical simulations.

## Introduction

Deep brain stimulation (DBS) is an effective therapy for systematic treatment of symptoms of movement disorders such as essential tremor, dystonia, and Parkinson’s disease (PD) [1]–[5]. In PD patients, electrodes are surgically implanted in the basal galia, usually in the subthalamic nucleus, for electric-pulse-generated stimulation of a population of neurons [6]–[8]. The high-frequency electronic pulse-train applied to the brain is characterized by certain stimulation parameters such as amplitude, frequency, pulse-width, and slope. Given the complexity of neurological processes, the underlying DBS mechanism remains unclear and subject to debate [9]–[10].

Mathematical modeling of PD and the physiological structures and functions of the relevant areas of the brain have been addressed in the literature [11]–[12] to explore medication strategies. Such studies, however, provide a complex model of PD that nonetheless, due to missing model information and unknown biological processes, cannot be utilized for tremor control. Recently, a number of studies have analyzed the relation between DBS parameters and tremor characteristics [13]–[16]. The effects of stimulation frequency on intention and postural tremor, for essential tremor patients, were studied in [15]. In another recent work [16], the effect of stimulation amplitude on tremor was investigated to explore the feasibility of an electronic tremor-symptom-assessment system. Studies like these can be used to develop a simple mathematical model of tremor, which further can be applied to the design of a control law for stimulation parameter estimation and motor symptom control.

Controlled DBS can be understood as a form of functional DBS that can be used to modify the movement-to-movement stimulation signal to ensure a reasonable quality. On-off feedback-controlled high-frequency stimulation in the sub-thalamic nucleus was recently investigated [17], in which the concentration of glutamate at the neurotransmitter level in the rat brain was controlled to suppress Parkinson’s tremor. On this foundation, researchers have explored more advanced, artificial-neural-oscillator- and local-field-potential-based feedback control algorithms essential to the development of a controlled stimulation framework [18]–[19]. These studies reveal the importance of proper stimulation parameters for feedback control to ensure maximum tremor suppression, development of an adaptive functional DBS, provision of an easy and automatic stimulation parameter selection procedure, and enhancement of pulse-generator battery life.

Although advanced techniques [18]–[19] of controlled DBS can facilitate understanding of stimulation therapy mechanisms, biological restrictions limit their implementation. The neural-oscillator-based technique [18], utilizing muscle stimulation, is restricted to tremor control of a single joint, and cannot guarantee suppression of tremor in other joints of the body. In practice, DBS is preferred over muscle stimulation as a therapy for control of brain-disorder-associated tremor. The other feedback-stimulation technique [19] requires a larger-size electrode for measurement of local field potentials. However, as the objective of DBS is tremor suppression, measurement of tremor signal is much more relevant and meaningful as a tremor control strategy than measurement of local field potentials. Moreover, local field potentials can be noisy, due both to the large number of complex processes in the brain and the interaction of the time-varying stimulus signal. Also, owing to the fact that every individual has a different spectrum of local field potentials, the tracking reference generation for these potentials, required for the closed-loop stimulation methodology in [19], is another challenging issue. Even if a reference spectrum is developed for a neurological patient, the required local field potential spectrum can change due to the time-varying nature of brain processes.

This paper discusses a feedback control of postural and rest tremor by DBS parameter estimation that achieves the advantages of controlled electrical stimulation with a methodology enabling the development of an automatic clinical device. A simple linear mathematical oscillator-based model of tremor under DBS is derived by application of the results of a kinematic study [16]. The linear model is modified as a more general time-varying nonlinear model of tremor. The feasibility of a robust adaptive feedback-controlled stimulation scheme, based on the measurement of tremor signals rather than the local field potentials, and on electronic stimulation of the brain rather than of muscle, is proposed. The proposed feedback control strategy, by utilizing the standard Lyapunov theory, guarantees exact estimation of the stimulation amplitude. Based on the adaptive feedback estimation of stimulation amplitude, a framework for an automatic DBS device is provided by formulating an algorithm for estimation of DBS parameters. The results of the proposed methodology for control of tremor are provided under different conditions.

This paper is organized as follows. Section 2 describes the linear/nonlinear tremor model, the proposed control methodology, the algorithm for DBS estimation, and the results of the proposed techniques. Section 3 addresses the methods employed, including tremor model validation, the Lyapunov method, and the proof of the main results. Section 4 draws conclusions.

## Results and Discussion

### 2.1. Oscillator-based Linear Tremor Model

**Figure 1:**
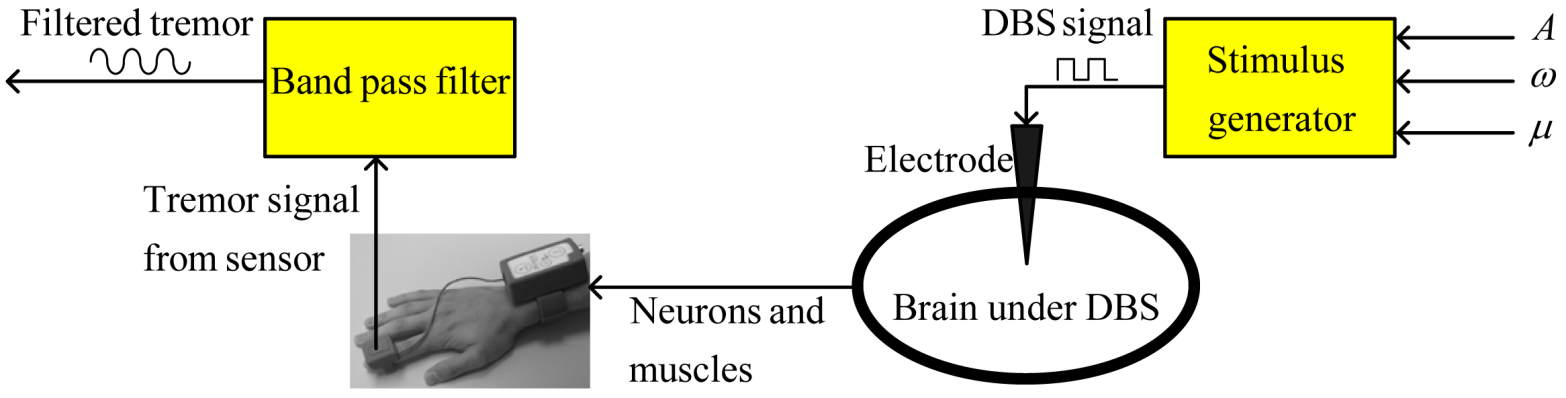
Process of deep brain stimulation (DBS) and tremor measurement. Values of DBS parameters are provided to the stimulus generator. The stimulus generator produces electrical impulses, which are applied to the brain through an electrode. The tremor signal generated at the hand and fingers is measured using a position, velocity or acceleration sensor (see [16]). This tremor signal can be filtered to isolate noise.

A DBS signal can be characterized by parameters such as stimulation amplitude *A*, frequency 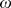, and pulse-width µ, which are provided to a stimulus generator to produce a stimulus signal. This stimulus signal is applied to a specific portion of the brain through an electrode in order to overcome the motor symptoms of a cognitive disorder. The tremor due to a brain disorder can be measured, in terms of acceleration, velocity or position, through a sensor attached to the finger of a patient. The noise in the tremor signal is removed by means of a band-pass filter. The entire process of tremor measurement and DBS is illustrated by the block diagram in Fig. 1.

Derivation of a mathematical model representing brain dynamics, neural circuitry and muscles, either for a healthy person or a brain-disorder patient, is very difficult, due to involvement of complex processes and some unknown biological mechanisms. Even if a dynamic model is derived to capture the properties of a tremor-based brain disorder (as in [12]), it cannot be utilized to control tremor by means of DBS, owing to model complexity and the significant amount of higher-order information on the brain that remains unknown. Such models, furthermore, are highly time-varying and vary also from case to case and person to person. For these reasons [12], it is almost impossible to design a feasible feedback control strategy for estimation of DBS parameters.

It is worth noting that the dynamics of the entire process shown in Fig. 1 can be represented by a simple time-varying oscillator, specifically by considering DBS parameters as inputs and the filtered tremor signal as the output. Many electronic oscillators have been used to represent a single neuron or cell [20]–[23]. Recently, a higher-order linear dynamic state-space model was developed to express the brain activity in response to neuronal activations [24]. In this light, utilization of an oscillator for representation of the overall brain and muscle dynamics of a tremor-oriented disease is remarkable for the simplicity of tremor control and the availability of the physics tools necessary for investigation of dynamic tremor behavior. It is notable that the filtered tremor signal is similar to a sinusoidal signal that can be characterized by its amplitude and frequency; therefore, tremor dynamics can be represented by means of a simple state-space model. For simplicity, the tremor dynamics can be described for a single input, which can be either of the stimulus parameters, for example, the amplitude. Consider the simplest second-order linear oscillator model of tremor dynamics in the state-space form, given by
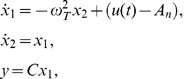
(1)where 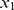 and 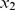 are the states of the oscillator, *y* represents the tremor, C and 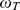 are time-varying parameters representing the strength of amplitude and the angular frequency of the tremor, respectively, and 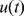 is the control signal for amplitude of the stimulus signal (generated by a feedback controller). The other parameters of stimulus, such as angular frequency 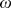 and pulse-width µ, are constant. It is assumed for model (1) that there exists a nominal value of stimulation amplitude, say *A_n_*, for which the tremor associated with a brain disorder vanishes (if the stimulation amplitude is selected as *A_n_*,) for given values of 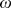 and µ. This assumption is reasonable, as the tremor of a patient with a brain disease like Parkinson’s is controlled by DBS for a certain selection of stimulation parameters [11]–[16]. Note that the tremor model (1) can be used to generate tremor signal closer to a sinusoidal function; therefore, it is applicable to filtered postural and rest tremor. To represent the dynamics of intention or action tremor, this model can be modified by incorporating additional states to represent variation from a sinusoidal function due to direction of movement.

The tremor model described by (1) is based on the fuzzy modeling approach utilizing the input-output information, that is, the idea of stimulation parameters and tremor characteristics. The main feature of the fuzzy modeling approach is that it does not require understanding of the whole process, such as functioning of the brain and mechanism of the DBS. The tremor output of any frequency and amplitude can be generated by selecting appropriate values of the parameters C and 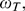 respectively. These parameters may depend on variations in neuronal signaling and behavioral changes in the brain, and we can study their values for different dynamical aspects of the brain by relating various works (such as [9]–[11] and [15]–[16]) on variation of tremor amplitude and frequency with respect to the brain mechanisms. For instance, to check the validity of the proposed linear model (1), the experimental results of [16] under different amplitudes of DBS corresponding to different neuronal behaviors are reproduced in Section 3 (see Subsection 3.1). Though, further studies can be undertaken for computation of the exact values and changes in the parameters under different dynamical aspects of the brain subjected to different stimulus conditions. For now, it can be stated that one of the main advantage of the linear oscillator-based tremor model is its utilization in designing a feedback controller for stabilization of tremor oscillations, for the purposes of which, computation of the exact values of model parameters C and 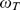 is unnecessary. By incorporating the known bounds on parameters C and 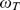, a feedback control methodology can be developed.

### 2.2. Oscillator-based Nonlinear Tremor Model

The oscillator-based linear tremor model can be extended by incorporating an additional term 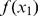 for the nonlinear aspect of the tremor-related state 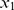. The tremor model (1) cannot be stabilized by an open-loop control (for 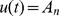) due to the lack of a stabilizing term. Therefore, a stabilizing term can be incorporated into tremor model (1) to obtain a more general tremor model. Hence, a nonlinear oscillator-based second-order tremor model can be written as
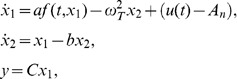
(2)where the parameters *a* and *b* represent the effects of nonlinear and stabilization terms, respectively.

A proper selection of nonlinearity 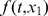 is needed for representation of tremor behavior. For instance, 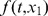 can be selected as 
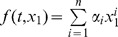
 for any positive integer *n*. Such selection of nonlinearity is more general than that in the traditional FitzHugh-Nagumo model where 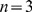 is utilized to represent the dynamics of biological oscillatory systems [20]. Another appropriate choice is the general Lipschitz nonlinearity [25]–[28], given its utility for representing many dynamic aspects of bio-systems and its tractable control law formulation for tremor. Such Lipschitz nonlinearities are often used to model engineering systems specifically called Lipschitz descriptor systems [29]. As motivated by engineering techniques, it is reasonable to select 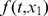 as a Lipschitz function in the first step. Additionally, such nonlinearity selection 
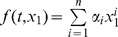
 also satisfies the Lipschitz condition in a local region. Therefore, we assume throughout the paper that the *unknown* nonlinearity 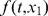 satisfies the Lipschitz bound below:
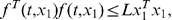
(3)where the scalar *L* is a positive constant. Details on the Lipschitz condition are provided in Section 3.

### 2.3. Adaptive Feedback Control

The purpose of the present study is to design a suitable feedback control law for stimulus amplitude 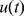 to stabilize tremor by utilizing the feedback of tremor signal *y*. The nonlinear tremor model (2)–(3) provides time-varying dynamics between control signal 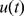 and filtered tremor signal *y*; therefore, it can be used to develop a control law for 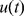 to suppress the motor symptoms. As a result, a new controlled DBS methodology can be developed by incorporating the knowledge of parametric bounds. To achieve robustness against variations in the model parameters, we make the following assumption.

#### Assumption 1

The parameters of the nonlinear tremor model (2)–(3) are bounded, that is, 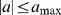, 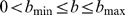, 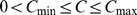, 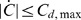, and 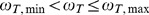, where 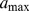, 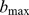, and 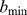 are bounds on the model parameters, 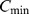 and 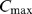 are associated with the minimum and maximum strengths of tremor amplitude, respectively, 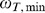 and 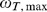 represent the minimum and maximum angular frequency of tremor, respectively, and 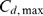 represents the maximum change in the strength of the tremor amplitude.

The following theorem develops a robust adaptive control scheme for estimating the DBS amplitude.

#### Theorem 1

Consider the nonlinear tremor model (2)–(3) satisfying Assumption 1. Suppose there exist scalars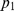, 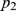, 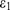 and 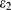, such that
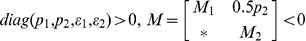
(4)are satisfied, where



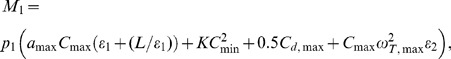
(5)

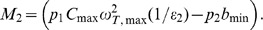
(6)


Then, the following control and adaptation laws
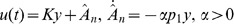
(7)ensure that

- *y* converges to zero, and

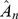
(and 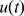) converges to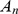, if the steady state (that is, convergence of *y* to zero) is achieved in a finite time.

#### Proof

The proof of Theorem 1 is provided in Section 3.

In practice, it has been observed that a system under an adaptive control law, such as in Theorem 1, can achieve its steady state in a finite time (see, for example [23]). In that case, the proposed adaptation law guarantees exact estimation of the nominal stimulation amplitude required for the stabilization of tremor.

Because of the safety requirement in biological systems, the control signal 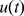 must be used as the stimulus amplitude after some necessary signal processing. For instance, if the amplitude of stimulation is within the 0∼5V range, the saturation nonlinearity
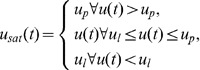
(8)can be introduced to limit the control signal. Typically, 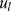 and 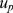 are selected as 0 and 5V, respectively. The saturated control signal 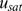 can contain high-frequency variations, which are not desirable in DBS. These variations can be isolated by means of a rate-limiter [30], given by
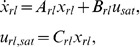
(9)where 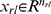 and 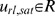 represent the state and output of the rate-limiter, respectively. The output 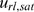 can be used as the stimulation amplitude under the condition of proper electrical isolation circuitry.

**Figure 2:**
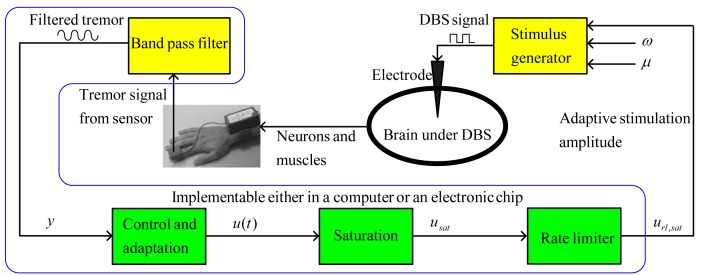
Proposed methodology for feedback control of tremor. The filtered tremor signal (see [16]) can be used to calculate the control signal using an adaptive control methodology. The amplitude of the control signal is bounded using a saturation block. The saturated control signal is passed through a rate-limiter to filter high-frequency variations in stimulus amplitude. The resultant signal can be used as the stimulation amplitude to generate a stimulus signal.

A block diagram of the overall closed-loop system is shown in Fig. 2. The blocks for noise filtration, control and adaptation, saturation, and the rate-limiter can be implemented using a single electronic processor. The proposed methodology for automatic estimation of DBS amplitude contains three units: a stimulation unit including a stimulus generator and electrode, a measurement unit with a tremor sensor, and an electronic processor comprising control, adaptation, and signal processing algorithms. It is noteworthy that the new feature of feedback-controlled DBS methodology will not significantly affect the size of the traditional stimulation devices, because it requires only an additional electronic processor chip for control, adaptation, and signal processing algorithms. The adaptive feedback control algorithm, given by (7)–(9), can be implemented in real-time for estimation of stimulation amplitude. Further, a clinical device framework can be developed to assist medical doctors in providing for automatic and more accurate estimation of DBS parameters (to be detailed in the following sub-section).

### 2.4. Algorithm for Stimulation Device

The main drawback of the adaptive methodology discussed in the previous sub-section is that it can be used for estimation of stimulation amplitude only if the other stimulation parameters are properly selected. For instance, if the parameters 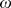 and 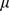 are selected improperly, estimation of *A* can be incorrect. To overcome this limitation, multiple estimations of stimulation amplitude can be performed for multiple values of stimulation frequency, say 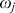 for 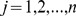, and stimulation pulse-width, 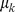 for 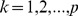. Each estimation can be performed for time 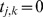 to 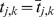, and the time-series of 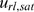 computed using (7)–(9) can be assigned to 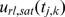. The corresponding 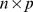 estimations of stimulation amplitude, denoted by 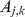, can be computed by taking the average of 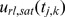 over an interval from 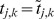 to 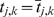, where 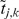 is the expected settling time in which 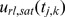 converges to a constant value. Out of a set of stimulation parameters given by 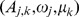 for 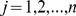 and 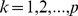, the appropriate stimulation parameters 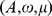 for which the tremor becomes minimum can be selected.

#### Algorithm 1

The step-wise procedure for the selection of stimulation parameters based on the proposed adaptive feedback control strategy is provided as follows:

1. Initialize the stimulation frequencies 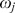 for 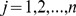 and the stimulation pulse-widths 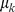 for 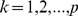.
2. For 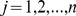

  For 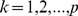.
  - Apply control law (7)-(9), from time 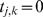 to 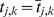, as shown in Fig. 2, by assigning 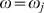 and 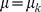, and compute the signal 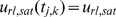.
  - Take the mean of 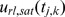 from 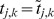 to 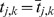 and assign it to 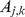. That is,

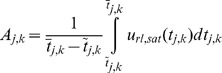
(10)
  - Measure the tremor signal *y* from time 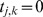 to 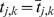 Calculate the root mean square of tremor (the tremor power), 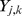, from 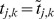 to 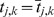, given by
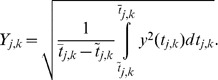
(11)End
    End
3. Find 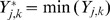 and the optimal set of stimulation parameters using

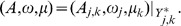
(12)
4. Apply DBS for the calculated stimulation parameters 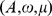 as in Fig. 1.

The main advantage of Algorithm 1 is that each 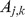 is selected from an infinite number of possibilities between 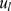 and 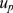, which can lead to a more reasonable selection of stimulation parameters. The optimal set of stimulation parameters, that is 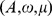, can be obtained by applying (12), which can be supplied to the DBS generator for stimulation purposes. Selection of parameters 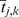 and 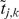 has an important role to play in algorithm performance, and this will be addressed in the next sub-section.

### 2.5. Results for the Proposed Feedback Control

The stimulation amplitude estimation controller is designed for 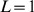, 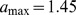, 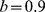, 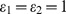, 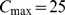, 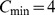, 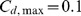, and 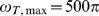. By solving the matrix inequalities of Theorem 1, the controller and the adaptation law parameters are obtained as 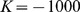, 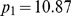 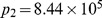, and 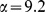. The saturation and rate-limiter parameters are fixed to 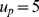, 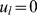, 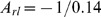, 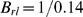 and 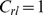. To evaluate the performance of the proposed methodology, we consider the two cases addressed below.

#### Case I

**Figure 3:**
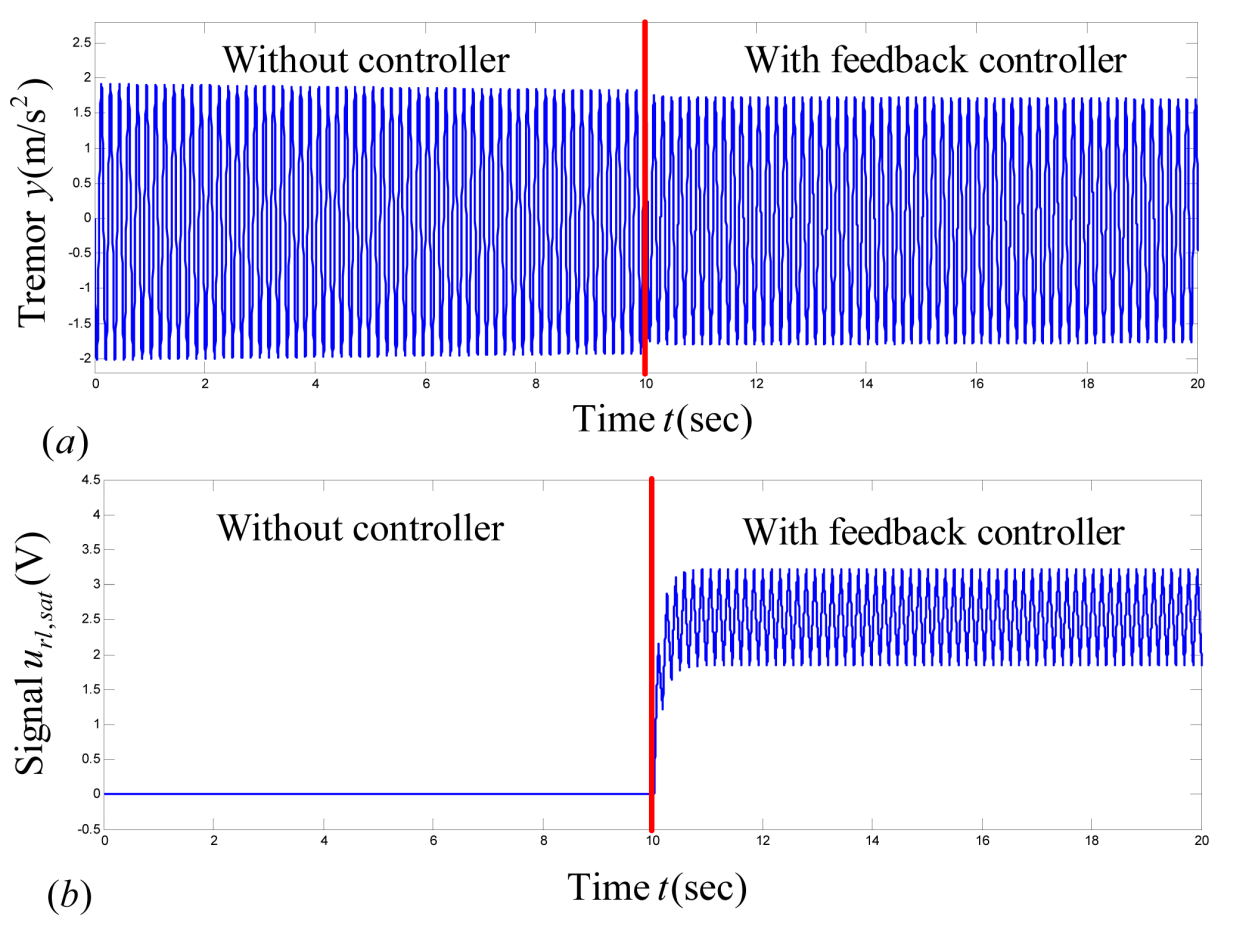
Closed-loop response of tremor by application of the proposed adaptive control methodology under Case I. The controller is applied at 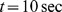. By application of the proposed controller, tremor 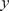 does not converge to zero, because a proper value of nominal stimulation amplitude 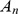 does not exist for this case. (a) Plot of tremor signal 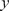, (b) plot of 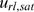, which does not converge to 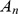 due to saturation.

First, we assume that for a selection of stimulation parameters 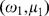, a proper value of nominal stimulation amplitude 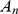 does not exist. For this reason, we can select 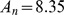 (that is, 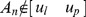) such that 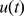 cannot converge to 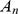 due to saturation. The remaining tremor model parameters are selected as 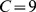, 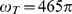, 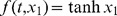, and 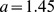 for time 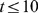, and 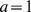 for time 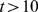. Fig. 3 shows the result of the proposed methodology for tremor and the applied control signal. The controller is applied at time 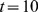 (that is, 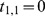 when 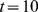). By application of the proposed methodology, the tremor does not vanish, because the assumption of existence of a proper value of 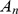 is not valid for this case, and the signal 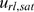 does not converge to 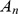. By applying (10)–(11), 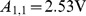 and 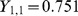 are calculated for 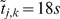 to 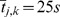. Tremor power 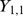 is not close to zero; therefore, 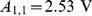 is not a suitable choice for DBS.

Due to the violation of the assumption on 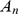 for selection of stimulation parameters 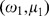, the proposed control methodology failed to estimate the desired stimulation amplitude. However, the solution to this problem is provided in Algorithm 1. Accordingly, we can apply another set of stimulation parameters, as in the following Case II.

#### Case II

**Figure 4:**
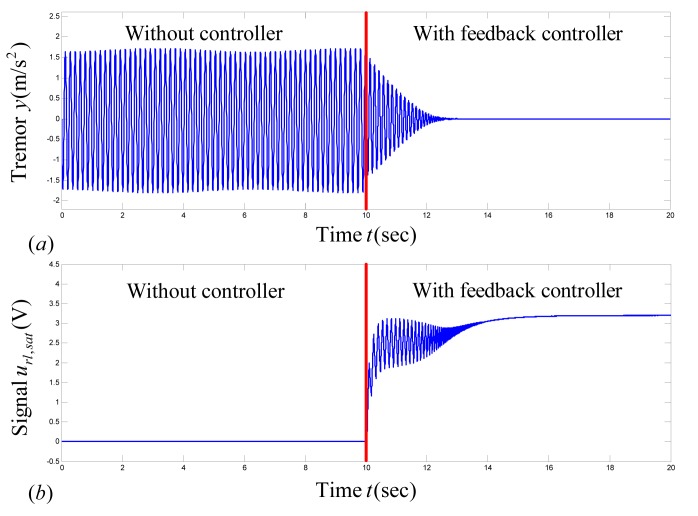
Closed-loop response of tremor using the proposed adaptive feedback-control methodology in Case II. The controller is applied at 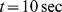. By application of the proposed controller, tremor 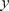 converges to zero, because a proper value of nominal stimulation amplitude 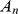 exists in this case. Hence, the proposed methodology by Algorithm 1 can be used for estimation of DBS parameters. (a) Plot of tremor signal 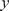, (b) plot of 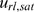, which converges to 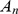.

Now we assume that the stimulation frequency has been changed from 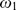 to 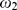, without changing the stimulation pulse-width, and that there exists a proper value of nominal stimulation amplitude, say 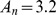, for 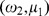. The model parameters are selected as 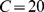, 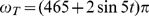, 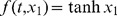, and 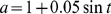. Fig. 4 shows the time-series for tremor and the control signal under saturation and the rate-limiter. As the controller is applied at 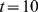 (with 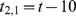), the tremor amplitude converges to zero and the signal 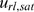 converges to 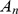. The mean value and the tremor power are calculated as 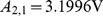 and 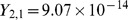, respectively, for 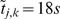 to 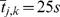. Clearly, 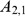 is closer to 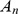 and 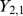 is nearly zero; therefore, 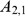 is a good estimate of stimulation amplitude. Hence, the proposed control scheme can be useful for adaptive estimation of stimulation amplitude.

**Figure 5:**
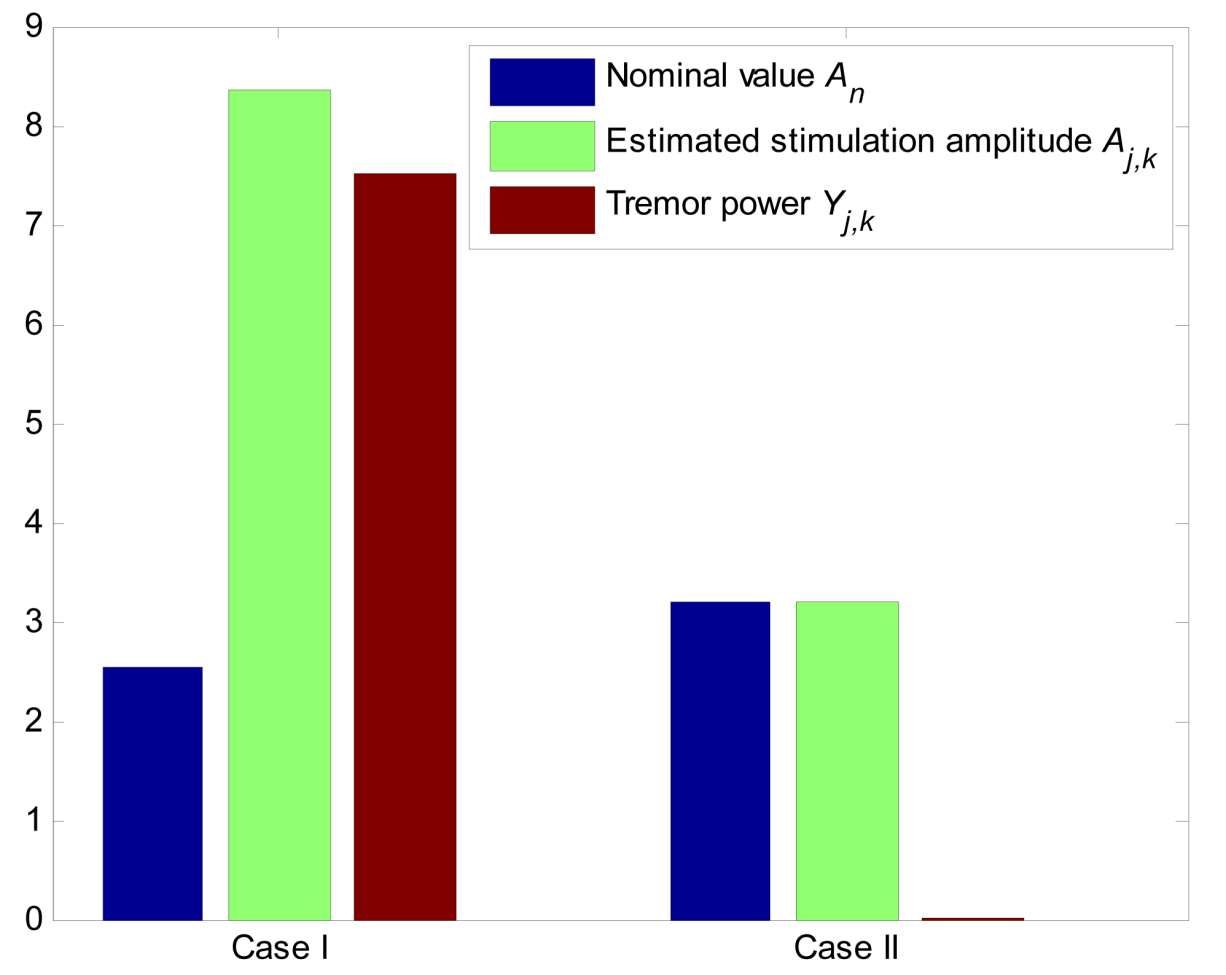
Comparison of estimation error and tremor power in Cases I and II. In Case I, the difference between 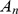 and its estimation is higher. Correspondingly, the tremor power is also higher. In Case II, the estimation of nominal stimulation amplitude 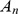 is correct; therefore, the tremor power is nearly zero.

By applying Algorithm 1, we can select 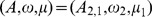 as simulation parameters. A comparison of tremor power and stimulation amplitude for Cases I and II is provided in Fig. 5. Such a diagram can be helpful when a number of selections for stimulation frequency and pulse-width are used to find appropriate stimulation parameters.

**Figure 6:**
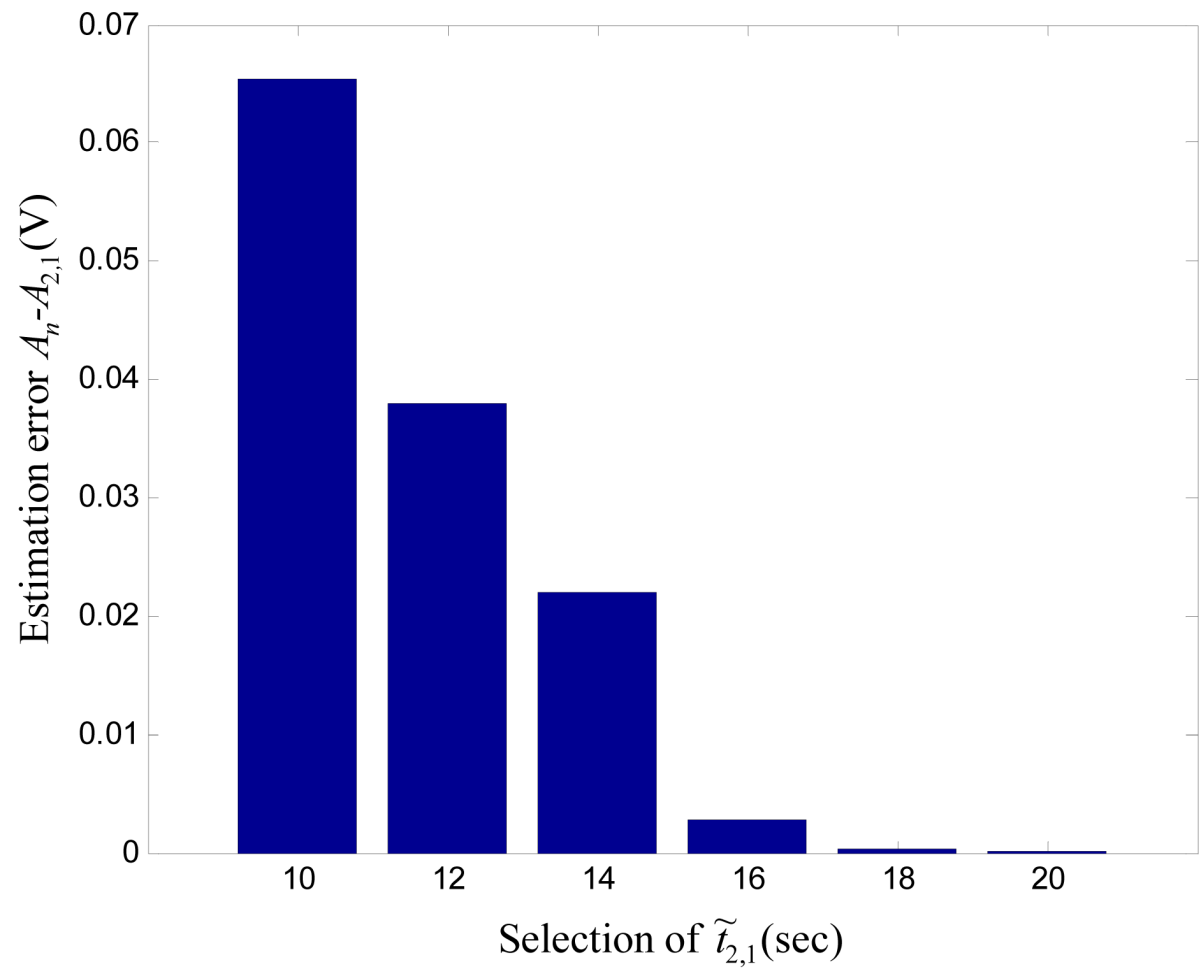
Effect of 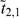 on estimation error 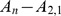 under constant 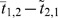. The estimation error is decreasing with increasing 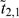. However, more time for estimation is required for higher values of 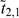. Hence, selection of 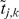 has critical effects on estimation time and estimation error.

Selection of 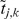 in Algorithm 1 is a critical issue. We now analyze the effect of 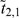 on 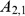 for Case II under the fixed value of 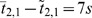. Fig. 6 shows the bar diagrams for 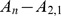 under different selections of 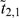. It is evident that the difference 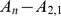 is decreasing with increasing 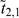. Hence, the selection of 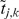 can play an important role in stimulation amplitude estimation. Better estimation can be obtained for a large value of 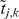, though the estimation time will be increased.

## Methods

**Ethic Statement:** N/A.

### 3.1. Linear Model Validation

**Figure 7:**
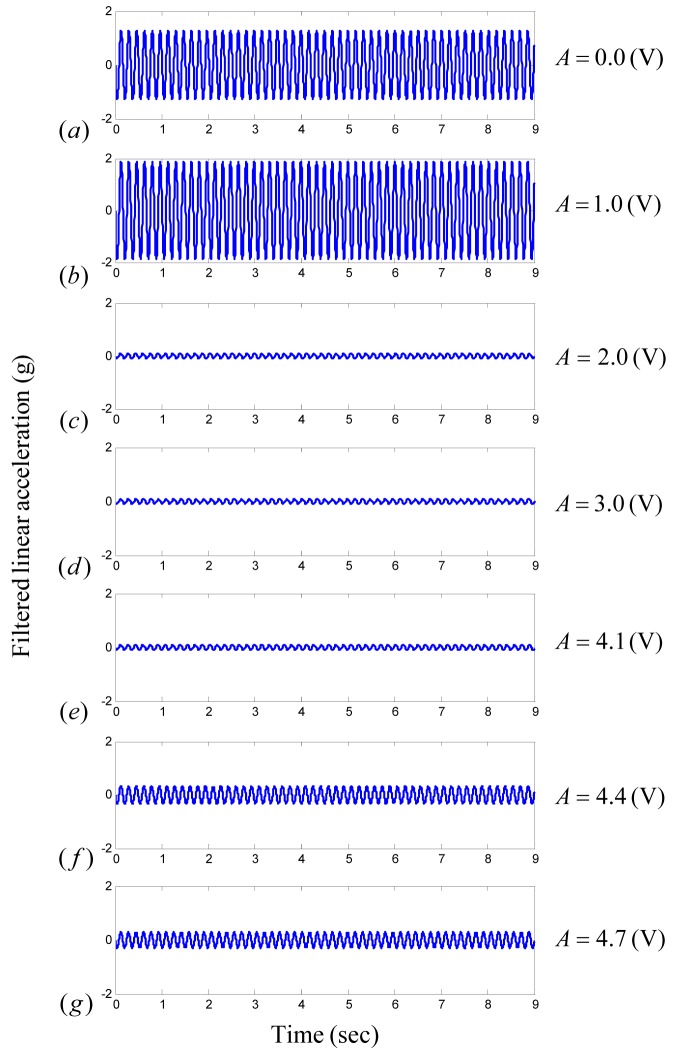
Filtered tremor acceleration under different values of stimulation amplitude. By considering time-varying parameters of the linear oscillator-based tremor model, we can reproduce the experimental results. In the present case, the results in [16] on the tremor of a patient under DBS with constant stimulation frequency and pulse-width are reproduced for different values of stimulation amplitude. The selected parameters of the model are as follows: (a)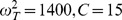, (b) 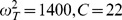, (c) 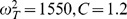, (d) 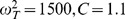, (e) 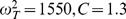, (f) 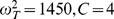, (g) 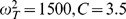.

The effect of DBS amplitude on tremor under constant stimulus frequency and pulse-width was studied in [16]. In Fig. 7, the Fig. 2(a) results in [16] are reproduced, for the same values of stimulation amplitude, by using the proposed oscillator-based linear tremor model (1). The nominal amplitude is taken to be 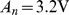. It is evident from Fig. 7 that the experimental results are reproduced for different values of parameters C and 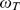, which validates the proposed oscillator selection for modeling of neurological disorder tremor.

### 3.2. Lipschitz Condition

A function 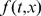 is said to be Lipschitz if it satisfies condition [25]–[28], given by
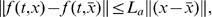
(13)Where 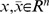, 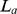 is a positive Lipschitz constant, and 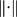
denotes the Euclidian norm. Note also that 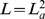. This condition can be used to derive a control law for Lipschitz nonlinear systems. Moreover, the Lipschitz nonlinearity also satisfies
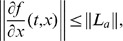
(14)which inequality can be useful in determining 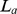 by means of a numerical algorithm. It is also important to note that if 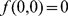, condition (14) becomes
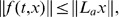
(15)which further simplifies the control law derivation. For a piecewise continuous function 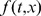 satisfying the Lipschitz condition, a unique solution to the differential equation 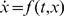 is guaranteed [25]. A function 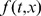 is said to be locally Lipschitz if it satisfies conditions (13)–(14) locally for a bounded region 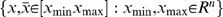, where 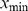 and 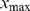 are the minimum and maximum limits on x, respectively. For further details, readers are encouraged to study the literature (e.g., [25]) on systems with locally and globally Lipschitz nonlinearities.

### 3.3. Lyapunov Stability

The Lyapunov method is used to study the stability and control of dynamic systems. Consider a dynamic system given by 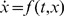, where 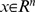 represents the state of the system. Suppose there exists a Lyapunov function 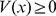 for all values of 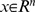 with equality only for 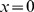
(that is, positive definite). If the derivative of the function 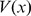 along the system 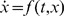 is less than or equal to zero (negative definite), that is, 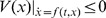 with equality only for 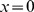 the state *x* will asymptotically converge to zero according to the Lyapunov theory (see, for example, [25]). The Lyapunov method can be applied to dynamic systems without any physical-energy requirement, and therefore is a valuable stability assessment tool.

### 3.4. Proof of Theorem 1

Incorporating control law (7) into (2), the following closed-loop system is obtained:
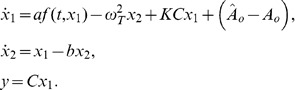
(16)


Consider a Lyapunov function
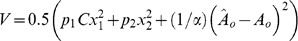
(17)


The time-derivative of Lyapunov function (17) along (16) is given by
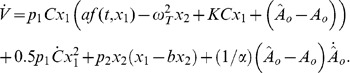
(18)


Incorporating the adaptation law (7) into (18), we obtain
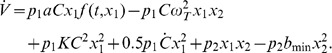
(19)


For positive scalars 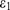 and 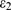, the inequalities
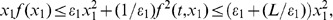
(20)

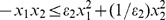
(21)


are satisfied. Incorporating these into (19), we obtain
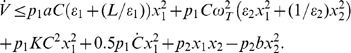
(22)


Using Assumption 1, it is implicit to obtain
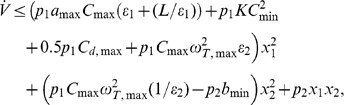
(23)which can be rewritten 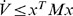, where 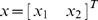 and *M* is given by (4)–(6). For stability 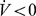 therefore, the matrix inequalities of Theorem 1 ensure that the tremor *y* converges to zero as 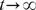, which completes the proof of statement (i) in Theorem 1. Now suppose that *y* converges to zero in a finite time 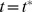. In the steady-state, 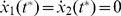, 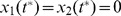, and 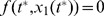. By means of the steady-state analysis for the closed-loop system (8), similarly to the work in [23], we obtain 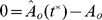. Hence, the adaptive parameter 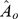 as well as the control signal 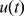 converges to 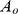, if the steady state is achieved in a finite time. This completes the proof of Theorem 1.

## Conclusions

In this paper, modeling and control of tremor under DBS was examined to explore the potentialities of an automatic feedback-controlled functional DBS scheme. An oscillator-based tremor model was developed in accordance with the linear systems theory by utilizing input-output knowledge. This linear model was extended to a more general tremor model by incorporating the nonlinearity and stabilization terms. By means of the standard Lyapunov theory, a robust adaptive feedback control methodology for estimation of the desired stimulation amplitude for tremor control was derived. Under the assumption of finite-time convergence of tremor, the proposed control methodology can be used to estimate the nominal value of stimulation amplitude required to control tremor. Based on multiple estimations of the nominal values for different stimulation frequency/pulse-width sets, an algorithm was established by using the proposed control methodology to derive the developmental framework of an automatic clinical device for functional DBS. The results of the proposed methodology were verified through numerical simulations of two cases.

## References

[1] Perozzo P, Rizzone M, Bergamasco B, Castelli L, Lanotte M, et al (2001) Deep brain stimulation of the subthalamic nucleus in Parkinson's disease: comparison of pre- and postoperative neuropsychological evaluation. Journal of the Neurological Sciences 192: 9–15. 11701147 10.1016/s0022-510x(01)00575-5

[2] Limousin P, Torres IM (2008) Deep brain stimulation for Parkinson’s disease. *Neurotherapeutics* 5: 309–319. 18394572 10.1016/j.nurt.2008.01.006 PMC5084172

[3] Jobst B (2010) Brain stimulation for surgical epilepsy. Epilepsy Research 89: 154–161. 19766458 10.1016/j.eplepsyres.2009.08.017

[4] Ostrem JL, Starr PA (2008) Treatment of dystonia with deep brain stimulation *Neurotherapeutics* . 5: 320–330. 10.1016/j.nurt.2008.01.002 PMC5084173 18394573

[5] Melzer N, Hicking G, Göbel K, Wiendl H (2012) TRPM2 cation channels modulate T cell effector functions and contribute to autoimmune CNS inflammation. PLoS ONE 7(10): e47617. 23077651 10.1371/journal.pone.0047617 PMC3470594

[6] Benabid AL (2003) Deep brain stimulation for Parkinson’s disease, Current Opinion in Neurobiology. 13: 696–706. 10.1016/j.conb.2003.11.001 14662371

[7] Wojtecki L, Elben S, Timmermann L, Reck C, Maarouf M, et al (2011) Modulation of human time processing by subthalamic deep brain stimulation. PLoS ONE 6(9): e24589. 21931767 10.1371/journal.pone.0024589 PMC3171456

[8] Frankemolle AMM, Wu J, Noecker AM, Voelcker-Rehage C, Ho JC (2010) Reversing cognitive–motor impairments in Parkinson’s disease patients using a computational modelling approach to deep brain stimulation programming. Brain 133: 746–761. 20061324 10.1093/brain/awp315 PMC2842509

[9] Modolo J, Beuter A (2009) Linking brain dynamics, neural mechanisms, and deep brain stimulation in Parkinson's disease: an integrated perspective. Medical Engineering & Physics 31: 615–623. 19243986 10.1016/j.medengphy.2009.01.005

[10] Bartolić A, Pirtošek Z, Rozman J, Ribarič S (2010) Tremor amplitude and tremor frequency variability in Parkinson’s disease is dependent on activity and synchronisation of central oscillators in basal ganglia. Medical Hypotheses74: 362–365. 10.1016/j.mehy.2009.06.057 19656636

[11] Titcombe MS, Glass L, Guehl D, Beuter A (2001) Dynamics of Parkinsonian tremor during deep brain stimulation. Chaos 11: 766–773. 12779515 10.1063/1.1408257

[12] Haeri M, Sarbaz Y, Gharibzadeh S (2005) Modeling the Parkinson's tremor and its treatments. Journal of Theoretical Biology 236: 311–322. 15950988 10.1016/j.jtbi.2005.03.014

[13] Beuter A, Titcombe MS (2003) Modulation of tremor amplitude during deep brain stimulation at different frequencies. Brain and Cognition 53: 190–192. 14607145 10.1016/s0278-2626(03)00107-6

[14] Ushe M, Mink JW, Revilla FJ, Wernle A, Gibson PP, et al (2004) Effect of stimulation frequency on tremor suppression in essential tremor. Movement Disorders 19: 1163–1168. 15390071 10.1002/mds.20231

[15] Earhart GM, Hong M, Tabbal SD, Perlmutter JS (2007) Effects of thalamic stimulation frequency on intention and postural tremor. Experimental Neurology 208: 257–263. 17920589 10.1016/j.expneurol.2007.08.014 PMC2203380

[16] Mera T, Vitek JL, Alberts JL, Giuffrida JP (2011) Kinematic optimization of deep brain stimulation across multiple motor symptoms in Parkinson's disease. Journal of Neuroscience Methods 198: 280–286. 21459111 10.1016/j.jneumeth.2011.03.019 PMC3122330

[17] Behrend CE, Cassim SM, Pallone MJ, Daubenspeck JA, A. Hartov A (2009) Toward feedback controlled deep brain stimulation: Dynamics of glutamate release in the subthalamic nucleus in rats. Journal of Neuroscience Methods 180: 278–289. 19464518 10.1016/j.jneumeth.2009.04.001

[18] Zhang D, Poignet P, Widjaja F, Ang WT (2011) Neural oscillator based control for pathological tremor suppression via functional electrical stimulation. Control Engineering Practice 19: 74–88.

[19] Santaniello S, Fienga G, Glielmo L, Grill WM (2011) Closed-loop control of deep brain stimulation: a simulation study. IEEE Transactions on Neural Systems and Rehabilitation Engineering 19: 15–24. 20889437 10.1109/TNSRE.2010.2081377

[20] Thompson CH, Bardos DC, Yang YS, Joyner KH (1999) Nonlinear cable models for cells exposed to electric fields I: General theory and space-clamped solutions. Chaos Solitons Fractals 10: 1825–1842.

[21] Aguilar-Lópex R, Martínez-Guerra R (2008) Synchronization of coupled Hodgkin-Huxley neurons via high order sliding-mode feedback, Chaos Soliton & Factals. 37: 539–546.

[22] Donnell P, Baigent SA, Banaji M (2009) Monotone dynamics of two cells dynamically coupled by a voltage-dependent gap junction. Journal of Theoretical Biology 261: 120–125. 19627994 10.1016/j.jtbi.2009.07.012

[23] Rehan M, Hong KS (2011) LMI-Based robust adaptive synchronization of FitzHugh-Nagumo neurons with unknown parameters under uncertain external electrical stimulation. *Physics Letters A* 375: 1666–1670.

[24] Aqil M, Hong KS, Jeong MY, Ge SS (2012) Detection of event-related hemodynamic response to neuroactivation by dynamic modeling of brain activity. Neuroimage 63: 553–568. 22796989 10.1016/j.neuroimage.2012.07.006

[25] H.K. Khalil, Nonlinear Systems, 3rd ed., Prentice Hall, New Jersey, 1996.

[26] Lu G, Ho DWC (2006) Full-order and reduced-order observers for Lipschitz descriptor systems: the unified LMI approach, IEEE T. Circuits -II. 53: 563–567.

[27] Lin W (2008) Adaptive chaos control and synchronization in only locally Lipschitz systems. Physics Letters A 372: 3195–3200.

[28] Rehan M, Hong KS, Ge SS (2011) Stabilization and tracking control for a class of nonlinear systems. Nonlinear Analysis: Real World Applications 12: 1786–1796.

[29] Abbaszadeh M, Marquez HJ (2011) A generalized framework for robust nonlinear H_∞_ filtering of Lipschitz descriptor systems with parametric and nonlinear uncertainties. Automatica 48: 894–900.

[30] Rehan M, Ahmed A, Iqbal N (2010) Design and implementation of full order anti-windup with actuator amplitude rate-limiter for an AC motor speed control system. Journal of the Chinese Institute of Engineers 33: 397–404.

